# Surgical osteochondral defect repair in the horse—a matter of form or function?

**DOI:** 10.1111/evj.13231

**Published:** 2020-02-19

**Authors:** Maria C. Fugazzola, Paul R. van Weeren

**Affiliations:** ^1^ Department of Equine Sciences Utrecht University Utrecht the Netherlands

**Keywords:** horse, cartilage, osteoarthritis, joint, surgical repair

## Abstract

Focal cartilaginous and osteochondral lesions can have traumatic or chondropathic degenerative origin. The fibrocartilaginous repair tissue that forms naturally, eventually undergoes fibrillation and degeneration leading to further disruption of joint homeostasis. Both types of lesion will therefore eventually lead to activity‐related pain, swelling and decreased mobility and will frequently progress to osteoarthritis. Most attempts at realising cartilage regeneration have so far resulted in cartilage repair (and not regeneration). The aim of this article was to review experimental research on surgical cartilage restoration techniques performed so far in equine models. Currently available surgical options for treatment of osteochondral lesions in the horse are summarised. The experimental validity of equine experimental models is addressed and finally possible avenues for further research are discussed.

## 1 INTRODUCTION

Focal cartilaginous and osteochondral lesions can have traumatic or chondropathic degenerative origin. The fibrocartilaginous repair tissue that forms naturally, eventually undergoes fibrillation and degeneration leading to further disruption of joint homeostasis.^1^ Both types of lesion will therefore ultimately lead to activity‐related pain, joint effusion and decreased mobility, frequently progressing to osteoarthritis.^2^ William Hunter's statement made in 1743 of articular cartilage being a tissue that ‘*when destroyed, it is never recovered*’^3^ is still applicable. In terms of ‘recovery’ of tissues, a distinction should be made between tissue regeneration and tissue repair. Regeneration refers to healing in which there is regrowth of tissue towards the original, normal state. In repair, there is a combination of regeneration and replacement by laying down connective tissue, mostly referred to as scarring.^4^ Most, if not all, attempts at realising cartilage regeneration have so far resulted in cartilage repair, not far from what endogenous repair would achieve in a joint.

**Figure 1:**
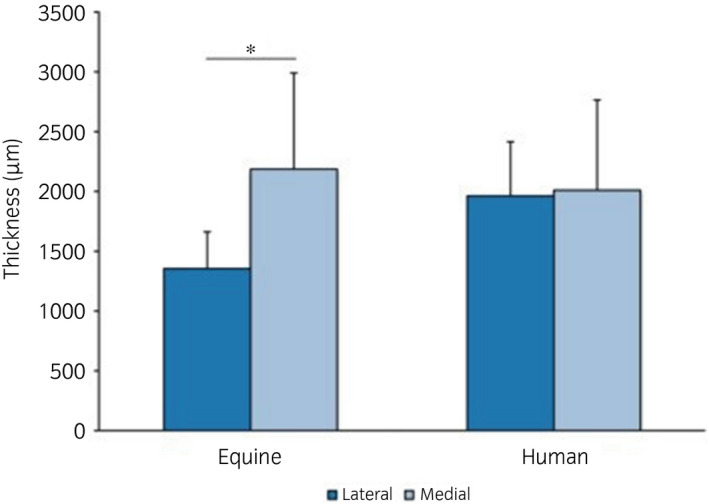
Average thickness of equine (n = 15) and human (n = 23) cartilage from the central areas of femoral condyles. Whereas a significant difference was observed between the lateral and medial condyle of equine samples (*P* = .003) in contrast to human samples, overall thickness is comparable. Error bars indicate 95% confidence intervals^1^

Compared to other animal models, articular cartilage thickness and subchondral bone thickness in the stifle of adult horses most closely approximates that of the human knee^5^, ^6^ (Figure 1). The horse is an athlete that suffers from similar debilitating cartilage lesions as human patients and therefore, in addition to being a patient in its own right, the horse is a model for the human patient. Because defects of relatively large size can be made experimentally in the horse, more outcome parameters (arthroscopic re‐evaluation, histological assessment, biomechanical testing, diagnostic imaging, biochemical analysis) can be measured with each repair response than is possible in other animal models.^7^ Based on two fundamental studies on spontaneous cartilage healing of experimental lesions in the equine stifle, the clinically relevant size for created osteochondral lesions has been determined to be 9 mm in diameter.^8^, ^9^ In the medial femoral condyle this corresponds to 15%‐20% of the weightbearing surface.^10^ For the intercarpal joint a more recent study defined 4 mm in diameter as the critical size for osteochondral lesions.^11^ These critical defect sizes refer to filling of the defect with repair tissue, not to tissue regeneration.

The aim of this article was to review high‐quality experimental research on surgical cartilage restoration techniques in the horse. Currently available surgical options for treatment of osteochondral lesions in the horse and their experimental validity are summarised. Applicability to human patients and the validity of equine models is addressed and finally possible avenues for further research are discussed.

## 2 EXPERIMENTAL STUDIES ON SURGICAL TECHNIQUES

The experimental studies discussed below are summarised in Table S1.

### 2.1 Microfracture

Marrow stimulation techniques, in particular microfracture, are routinely used for full‐thickness defects with an intact subchondral bone plate in horses.^12^ This procedure is believed to stimulate endogenous cartilage repair and to facilitate influx of stem cells and growth factors that originate from beneath the subchondral bone plate. Three basic research studies on microfracture in the horse in the medial femoral condyle and in the radial bone of the carpus have been performed, but only one is a long‐term study (12 months). Lesions treated with microfracture showed more defect filling when compared with no treatment in terms of quantity of repair tissue.^10^, ^13^, ^14^ Histologically, composition of repair tissue, including the relative presence of collagen type 2, was not different between lesions treated with microfracture and untreated lesions. Functionality in terms of biomechanical strength of the repair tissue was not assessed in any of these studies. In the human field, microfracture has been questioned, because studies supporting effectiveness are mainly derived from case series and there are few randomised trials.^15^ A large systematic review on microfracture for the treatment of osteochondral defects in the knee in human patients showed that in most cases clinical outcomes improved with microfracture at short‐term, but in some studies and over the longer term these effects were not sustained.^16^ One of the negative outcomes appears to be the formation of intralesional osteophytes.^17^, ^18^, ^19^, ^20^ This might represent further degeneration of repair fibrocartilage triggering a reactivation of the endochondral ossification mechanism once the subchondral bone plate is perforated.^21^ The phenomenon has also been seen in equine studies in which chondral defects were treated with microfracture and concentrated bone marrow aspirate.^22^ In human patients, the quality of cartilage repair following microfracture is variable and inconsistent for unknown reasons and younger patients have better clinical outcomes and quality of cartilage repair than older patients.^23^, ^24^ Reasons for variation in clinical outcome after microfracture remain unclear but potentially may be explained by factors such as preexisting inflammation or genetic predisposition and there is limited evidence that microfracture should be accepted as gold standard for the treatment of cartilage lesions in the knee joint.^15^ Nevertheless, the technically simple and inexpensive nature of this treatment makes microfracture a popular treatment for chondral and subchondral articular lesions in human and equine patients.

### 2.2 Mosaicplasty

Arthroscopic mosaic arthroplasty or mosaicplasty is commonly used in human surgery to repair large chondral defects by harvesting osteochondral cores from nonweightbearing areas and transplanting these to the affected site.^25^ This approach has been evaluated experimentally in the equine carpus and stifle. In one study three osteochondral grafts were harvested arthroscopically from the femoropatellar joint and transplanted to the third carpal bone. At 9 months post‐operatively osteochondral grafts in the third carpal bone had less proteoglycans, leaving the cartilage softer and less resistant compared to surrounding cartilage. Six of 18 grafts had histological evidence of cartilage degeneration and it was suggested that discrepancy in cartilage thickness between donor and recipient site was a major limitation with this technique.^26^

**Figure 2:**
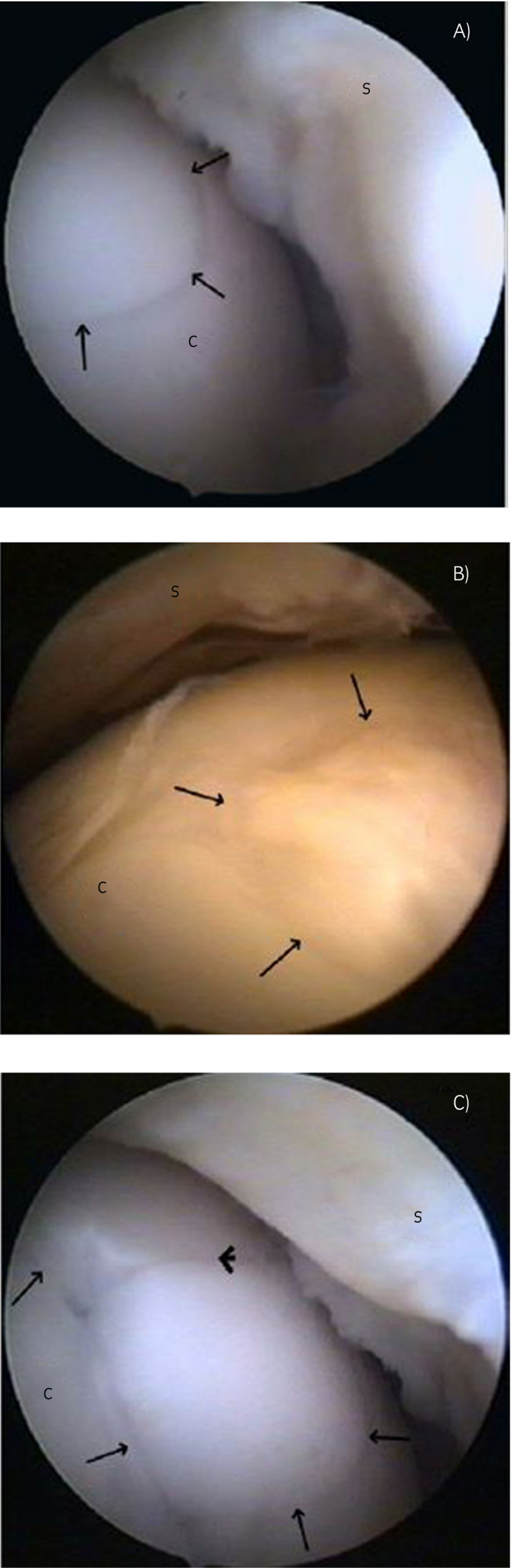
Follow‐up arthroscopic images of mosaicplasty recipient sites. A, The transition between the host bed and the transplant (arrows) is hard to recognise after 12 mo. B, A congruent but irregularly rippling surface of a transplant (arrows). C, Protuberance of the transplanted graft cap (arrows) and a small gap formation (arrowhead) at the interface region between host bed and transplant. S, synovial membrane. Reprinted with permission from Bodo et al*.* (2013) Mosaic arthroplasty of the medial femoral condyle in horses—an experimental study. *Acta Vet. Hung*. **62**, 155‐168

In another study, osteochondral plugs were harvested from the cranial surface of the medial femoral trochlea and implanted into defects on the weightbearing surface of the contralateral medial femoral condyle in five horses. After 12 months, 50% of the grafts showed hyaline cartilage, whereas the other half showed loss of glycosaminoglycans and transformation to fibrocartilage. During follow‐up arthroscopy at 12 months the transplanted areas looked smooth and congruent and radiologically there were no signs of osteoarthritis^27^ (Figure 2). Most donor sites were rebuilt with cancellous bone and covered by fibrocartilage and 3 out of 60 showed mild fibrillation of the surface.

Autologous chondrocyte implantation and matrix‐assisted autologous chondrocyte implantation.

Autologous chondrocyte implantation (ACI) and the more evolved matrix‐assisted autologous chondrocyte implantation (MACI) are frequently used for cartilage repair in human patients. The techniques involve harvesting chondrocytes from a nonweightbearing surface, expanding them in vitro and implanting them in a second surgical procedure. A single step procedure has also been described, first in an equine model and then applied in human patients.^28^, ^29^ In the equine stifle ACI secured with a periosteal flap and fibrin glue led to an overall improvement of histological scores compared to nongrafted defects but the repair tissue was not different in composition from fibrocartilaginous repair and the study had a short follow‐up period of only 8 weeks.^30^ Combining the ACI procedure with growth factors (IGF‐1) and using genetic overexpression of IGF‐1 and BMP‐7 stimulated early repair within the cartilage defect, but in the long‐term results were less significant.^31^, ^32^ Moreover, although total pathology scores from defects repaired with chondrocytes expressing IGF‐1 were significantly improved compared to naïve chondrocytes or fibrin filling alone, there was no significant difference between genetically manipulated chondrocytes expressing the IGF‐1 gene and the positive control group expressing a null gene (GFP),^32^ thus the benefit of IGF‐1 overexpression is questionable.

In MACI, chondrocytes are cultured and supported in a three‐dimensional environment. The membranes used for this purpose have a low friction coefficient on one side and permit chondrocyte infiltration on the other. The first MACI study in the horse was performed using a collagen membrane from porcine sub‐intestinal mucosa. After culturing chondrocytes on the membrane, this was implanted in defects on the medial trochlear ridge of the femur and fixed with polydioxanone/polyglycolic acid (PDS/PGA) staples.^33^ After promising early results, the same research group then compared a new single step surgical procedure using autologous cartilage fragments on a PDS scaffold, with a classic two‐step ACI technique. The ACI technique and the chondrocyte‐loaded polydioxanone scaffold were both associated with higher arthroscopic, immunohistochemical and histological scores than empty defects and defects with PDS foam alone.^28^ However, after a first human prospective clinical safety trial of 2 years with positive results in 2011, no further mention of this technique is to be found in the medical literature.^29^ The MACI technique has been evaluated in the horse using a collagen type 1/2 membrane as a scaffold to repair 15 mm diameter defects on the femoral trochlear ridge and a 6‐month follow‐up period. MACI showed better gross healing compared to empty defects and histological, histochemical and immunohistochemical scores were significantly better in treated compared to untreated defects within the same stifle. Mechanical proprieties of the MACI repair tissue were not different from repair tissue in control lesions, left to heal spontaneously.^34^

The same research group repeated the MACI experiment with longer duration (53 weeks) and a larger number of animals.^35^ The biomechanical proprieties of the repair tissue in this study were presented in a separate publication.^36^ Two chondral defects created on the lateral trochlear ridge of one stifle were treated with MACI, MACI membrane without chondrocytes, or left empty. Histological and immunohistochemical evaluation of defects treated with MACI showed overall improvement compared to empty defects. Biomechanical proprieties of the repair tissue from all treatment groups were compared to native cartilage of the same location from the control limb and the frictional properties of all implants were similar to control tissue. However, the compressive moduli of repair tissue in defects filled with MACI membrane alone (without seeded chondrocytes) and repair tissue from defects left empty had equilibrium moduli that were 46% and 59% of control respectively. The resistance to shear forces in all groups was also significantly different from native cartilage, being 4‐10 times lower in MACI‐treated defects than in control cartilage.^36^

The weak point of these MACI experiments is that they use an untreated chondral defect as control for arthroscopic and histological comparisons. Chondral defects in the horse will show little, if any, spontaneous healing^8^, ^11^ and improvement of healing in the sites treated with MACI in comparison with empty defects is therefore not very strong evidence of efficacy. Although generally seen as one of the best options for cartilage repair in human patients, it is still questionable whether MACI treatment should be considered superior to microfracture in the horse, especially when taking into account reduced cost and simpler nature of microfracture.

### 2.3 Mesenchymal stem cells and progenitor cells

The first experimental study using bone marrow aspirate for treatment of clinically relevant chondral defects in an equine model compared concentrated bone marrow aspirate concentrate (BMC) in combination with microfracture with microfracture alone. All outcome scores and magnetic resonance imaging supported improved healing in the bone marrow group but no biomechanical testing of the repair tissue was performed.^22^ Due to concerns about potential undesirable subchondral bone changes after microfracture, the study was repeated with a slightly different protocol and with a longer follow‐up (12 vs 8 months) to test the hypothesis that application of BMC without microfracture would improve repair compared to microfracture alone. However, BMC treatment resulted in fibrocartilage that was not different compared to the microfracture group.^37^ It was also shown that the quantity of mesenchymal stem cells in minimally manipulated BMC was not sufficient to generate cartilage pellets in vitro, which might explain the negative outcome in vivo. Qualitative MRI assessment showed improved subchondral bone characteristics in the BMC treated group compared with the microfracture group, but this finding can be considered trivial, as subchondral bone reaction will obviously be more evident if the subchondral bone plate is perforated.^38^

A study using bone marrow‐derived‐MSCs (BMSC) in fibrin for repair of full thickness articular defects in the lateral trochlear ridge of the femur had promising results at 1 month, but did not show significant differences at 8 months.^39^ In another study in the same model, BMSCs in a fibrin/platelet‐rich plasma (PRP) hydrogel showed inferior repair compared to the fibrin/PRP injected controls. In 4 out of 12 cases the BMC‐enriched fibrin/PRP defects was associated with bone formation within the defect.^40^

### 2.4 Allografts, autologous grafts and bioprinting

Artificial biological scaffolds can be manufactured in a more reproducible way than natural scaffolds and will also provoke less immune‐related problems. Hydrogel scaffolds for cartilage repair are being studied intensively. In an equine experimental study, an injectable self‐assembling peptide showed no improvement of repair tissue compared to microfracture alone, or to a combination of microfracture with the peptide. In fact, microfracture produced significantly better results in the histological and immunohistochemical evaluation of the repair tissue and in the histological evaluation of the synovial membrane when compared with all other treatment groups.^41^ Still, when tested biomechanically for dynamic, shear and static stiffness, control native cartilage was 10 times superior to the best repair tissue, highlighting the importance of biomechanical testing in cartilage repair research. Another finding was that samples of repair tissue from defects treated with microfracture, which were harvested from a more proximal region of the lesion, differed significantly in terms of histology and immunohistochemistry compared to samples from a more distal site. This observation suggests there is a need to consider the possible influence of biomechanical forces on repair outcomes depending on the location in the joint.

A dehydrated micronised allograft cartilage scaffold mixed with PRP was evaluated in an equine experimental model as a pre‐clinical trial for commercial use.^42^ The main conclusions from the study were that the implant was biocompatible and safe. Conclusions about effectiveness are more difficult, given the very small population size (n = 5) of the study. Tissue integration and collagen type 2 presence for one of the two treated defect locations were superior when compared with the control group which underwent microfracture alone. No biomechanical testing was performed on repair tissue.

### 2.5 Biphasic grafts and zonal constructs

With the exception of mosaicplasty, most equine experimental work has addressed chondral defects. Additional depth of the defect increases the level of complexity, as the barrier of the calcified layer disturbs endogenous.^10^ Further, when defects breach the osteochondral plate, lack of subchondral bone to support repair tissue is an additional problem in restoring functional repair. Research on equine osteochondral defect repair is recent (I Mancini et al, unpublished data, 2019). The concept of reproducing different layers of an engineered construct that mimics the multilayer structure of articular cartilage down to subchondral bone was first tested with a cartilage repair device consisting of a biphasic bioresorbable scaffold with a chondral and a subchondral phase. The implant was immersed in bone marrow aspirate and implanted in an osteochondral defect and compared to lesions treated with microfracture. In a 2‐year follow‐up there were no significant differences in histological scores of osteochondral and synovial tissue, or in biomechanical testing.^43^

**Figure 3:**
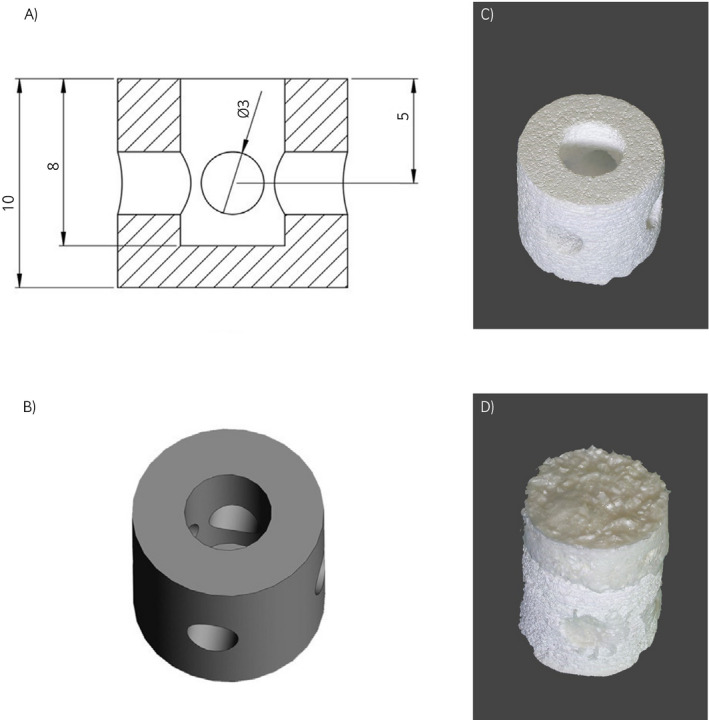
Macroscopic views of a composite osteochondral scaffold. A, The design of the mould in which the cartilage‐derived matrix (CDM) scaffold is cast on the printed calcium phosphate (CaP) scaffold (numbers indicate distances in mm). B, 3D design of the CaP scaffold. C, 3D printed CaP scaffold. D, 3D printed CaP scaffold with CDM cast on top

**Figure 4:**
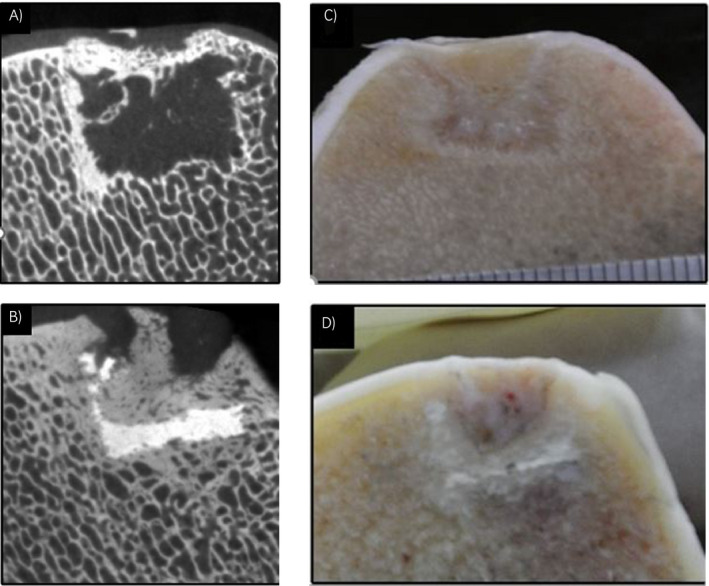
Micro‐CT and macroscopic pictures, respectively, of implantation sites at 6 mo. A) and C) created osteochondral defect treated with cartilage derived matrix (CDM) scaffold only: the void below the scaffold implant shows lacking bone repair. B) and D) created osteochondral defect treated with a combined CDM and Calcium phosphate scaffold: good integration of the scaffold within the bony portion of the defect^3^

A decellularised matrix scaffold has been tested first in a short‐ and subsequently in a long‐term equine model. A collagen‐derived matrix was implanted alone or combined with a 3D‐printed calcium phosphate cement‐based scaffold to fill osteochondral defects in the middle trochlear ridge of the femur of eight adult horses (Figure 3). The hypothesis that the composite scaffold would lead to overall better anatomical reconstitution and that the chondral portion would heal with repair tissue closely resembling hyaline cartilage could not be confirmed.^44^ After 6 months, histology and biochemistry showed predominantly fibrotic repair tissue, without significant differences between groups. The bony portion of the scaffold was, however, well integrated within the surrounding bone tissue (Figure 4).

### 2.6 A matter of form or function

The high number of equine ongoing studies concerning chondral and osteochondral defect treatment and the diversity of approaches used, is proof of a still missing long‐term solution. In fact, no technology has yet brought convincing evidence of efficacious long‐term cartilage repair. The reason for the relative stagnation of progress in the field may not only be technical in nature, but may also relate to more conceptual issues, such as the use of terminology and the choice of animal models.

**Figure 5:**
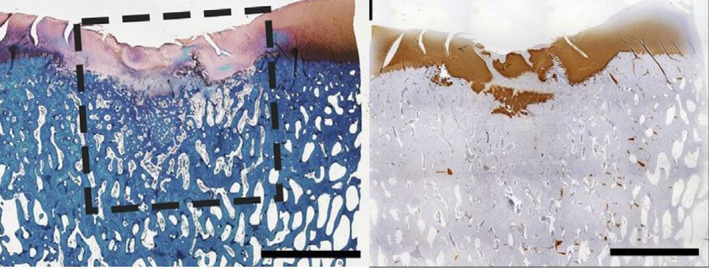
Histological view of an artificial defect treated with a decellularised cartilage derived matrix scaffold after 8 wk. Newly formed tissue was rich in glycosaminoglycans (stain red with safranin‐O) in the left image. Collagen type 2 stained brown in the right image. Scale bar represent 500 mm. Interrupted lines represent the outline of the artificial defect that was filled with the scaffold^3^

**Figure 6:**
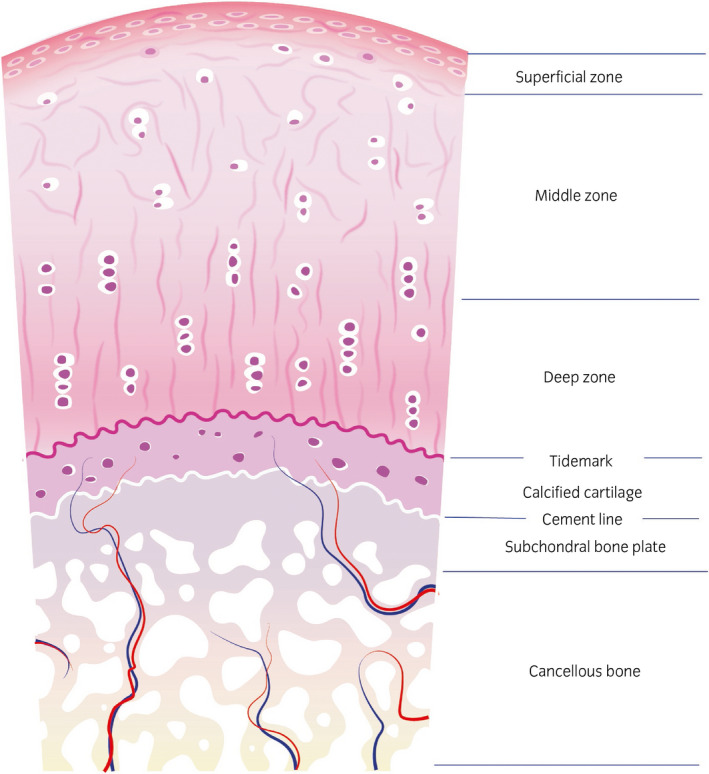
Schematic drawing of the structure of mature articular cartilage and subchondral bone. The tissue consists of a hyaline cartilage layer with three distinct zones: the superficial, middle and deep zone; the calcified cartilage layer and the subchondral bone

Positive results in the reported studies mostly refer to the presence of collagen type 2 or glycosaminoglycans (GAG) in the (immuno)histochemical analysis of the repair tissue (Figure 5). Although the presence of these components is obviously necessary as a first step, the appreciation of their presence within repair tissue should perhaps be reconsidered taking into account that (a) the amount of these components of articular cartilage is almost invariably less than in native cartilage and the distribution often different and that (b) none of the neo‐tissues thus far has shown any regeneration of the structural organisation of these components, or the tissue's architecture, which is indispensable for creating the right biomechanical properties. The zonal arrangement of collagen type 2 within the extracellular matrix in a very specific arcade configuration^40^ is a crucial biomechanical feature for resilience of mature native articular cartilage (Figure 6). Collagen structure and fibril orientation can be assessed through a number of novel technologies in a noninvasive manner.^45^, ^46^, ^47^ Fourier transform infrared microspectroscopy, polarised light microscopy and near infra‐red spectroscopy have been used in the equine joint to assess developmental changes during growth^46^ and to evaluate perilesional areas and the proprieties of the repair cartilage.^45^ The biomechanical function of cartilage relies on its composition but, perhaps more importantly, on the three‐dimensional structure of the collagen network. Therefore, assessment of these characteristics should be routinely included when evaluating the functionality of neo‐tissue that is formed. Most of the studies summarised above did not perform any form of structural assessment or biomechanical testing of the repair tissue and those which did, consistently showed that, regardless of the treatment modality, the repair tissue had poor biomechanical characteristics compared to native cartilage. The biomechanical strength of natural repair tissue, that is fibrocartilage, with its predominance of collagen type 1, might be biomechanically stronger or at least similar in biomechanical strength to repair tissue where collagen type 2 and GAGs are abundant but lack structural arrangement. The tensile strength of fibrocartilage as a natural constituent of the body (about 10 MPa) is less than that of tendon (about 55 MPa) but greater than that of hyaline cartilage (about 4 MPa).^48^ In compression, the strength of fibrocartilage is similar to that of hyaline cartilage, but it is less stiff. Both the aggregate and elastic moduli are about half those of articular cartilage.^49^, ^50^ Although early biomechanical studies were performed on other types of fibrocartilage than articular repair fibrocartilage, they give some insight in the properties of this tissue and question its inferiority when compared with presumed ‘better quality’ repair tissue, a qualification which, in most equine cartilage repair research, has been based solely on the histologically determined presence of Collagen type 2 and GAG production. The importance of the collagen network was exemplified in a study on the biomechanical consequences of progressive and selective enzymatic digestion of proteoglycans and collagen in the superficial layers of bovine articular cartilage.^51^ Digestion with collagenases made shear resistance of the superficial layer drop below detectable values while digestion of proteoglycans, with a still intact collagen network, kept that resistance almost nonaltered. The evaluation of mechanical proprieties of repair tissue in cartilage defects is therefore crucial to assess its long‐term performance. Earlier studies had already shown that shear properties of normal cartilage are highly dependent not only on collagen content but more specifically on collagen organisation.^52^ Hence, in the light of increasing evidence of the complex relationship between structure and function of repair cartilage, biomechanical and structural collagen assessments of any repair tissue should be considered indispensable if conclusions about clinical relevance are to be made.

### 2.7 Terminology

**Figure 7:**
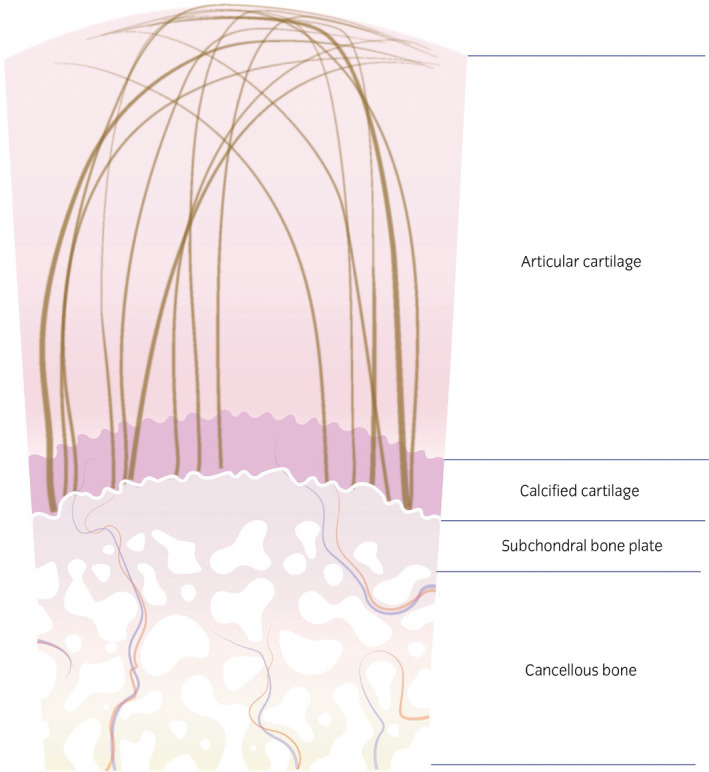
Schematic drawing of the arch‐like configuration of the collagen fibres in articular cartilage. Fibre orientation in the superficial zone is tangential to the cartilage surface, in the deep zone perpendicular to it. In the middle zone the fibres describe an arc, resulting in a nonparallel configuration

Reviewing the studies summarised above, it is noticeable that the terms hyaline cartilage, hyaline‐like cartilage and native cartilage are used in a confusing manner. Benninghoff in 1925^53^ clearly distinguished between articular hyaline cartilage and nonarticular hyaline cartilage with the sole but essential difference in terms of biomechanical properties being the arcade formation of collagen in hyaline cartilage of joints (Figure 7): He described an ‘*arrangement of continuous arcades which run radially in the middle of the cartilage, curving at the periphery to run parallel to the tissue boundary before returning into the tissue depth*’. The term hyaline cartilage or hyaline‐like cartilage has become a synonym for native cartilage but in research reports it is often used to refer to repair tissue containing collagen type 2 and GAGs. This use of the term subtly suggests that the repair tissue is equivalent to native cartilage, which it is evidently not. The mere presence of collagen type 2 and GAGs in repair tissue does not justify the term hyaline cartilage as a synonym of native cartilage. These repair tissues distinguish themselves from fibrocartilage only by variations in relative presence of collagen types and variable amounts of GAGs,^54^, ^55^ but are far off from native cartilage.

### 2.8 Equine experimental models

In terms of translation of findings from experimental studies summarised above to clinical practice, it is critical to recognise that all joints involved in the cited studies were ‘healthy’ to begin with. This is unlike the clinical situation and the influence of disrupted homeostasis of an inflamed joint or the degradation of subchondral bone, as frequently seen clinically, could obviously not be taken into account. This does not completely annihilate the value of the outcome, but it certainly is a limitation. The situation is not likely to change, as large‐scale randomised double‐blind controlled studies in horses with naturally occurring joint disease are very difficult to carry out.

Although not a perfect fit, the horse is considered one of the best animals for experimental research on cartilage repair. Many preclinical trials and preliminary work for commercialisation of products destined for the human or equine market have been based on successful outcomes in experimental work in rabbits. However, it has become clear that rabbits, mice and other small‐size mammalians are not a good translational species for studies on human or equine cartilage repair because of their small joint size, thin cartilage and greater potential for intrinsic healing than human patients or other large mammalian species.^56^ Therefore, although economically convenient and easy to manage, use of rabbits for osteochondral repair studies for human application, even if only as proof‐of‐concept, falls short of ethical good practice in animal experimentation and the three Rs principles.^57^ At present, the horse is recognised as one of the most appropriate animals for evaluation of cartilage repair strategies prior to human (and equine) clinical trials.^5^, ^6^, ^7^, ^58^ However, it should be recognised that, after 20 years of refining repair techniques in the horse, the overall success in long‐term cartilage repair is still very meagre. Cynically, it could be stated that this meagre outcome confirms the translational value of the equine model, but it also shows the model's challenging character. Immediate post‐operative weightbearing is unavoidable in this species, which subjects implants to a heavy challenge in the early recovery phase and much higher biomechanical forces are involved compared to those in any human post‐operative rehabilitation protocol. In the equine stifle joint, the most clinically relevant defect sites for the equine patient are the lateral trochlea and medial condyle of the femur. The first is a predilection site for osteochondrosis and the second for the development of subchondral cystic lesions.^59^ However, the lateral and medial trochlea of the femur are used most often as experimental sites, since they allow for relatively easy access. In human athletes 37% and 35% of focal chondral lesions are found in the femoral condyles and the femoro‐patellar joint respectively (of which 64% subpatellar and 36% on the trochlea).^60^ For this reason, it might be questioned whether the equine medial femoral condyle would not be a more pertinent location and should be preferred to the trochlear ridges. Further, compared to the femoropatellar joint, the more compressive rather than shear loading biomechanics of the femorotibial joint should be considered, as the type of loading is likely to influence the healing process.

Ovine and caprine models are also available. When compared with human patients, goats have similar advantages regarding cartilage and bone thicknesses as the horse, they have similar dimensions and anatomic relations as the human knee and might represent a less challenging environment in terms of immediate load bearing for a repaired osteochondral lesion.^61^, ^62^ Longitudinal monitoring by means of repeat arthroscopy, sequential sampling of synovial fluid and quantitative gait analysis, is more difficult than in the horse. Furthermore, these species lack one of the great advantages of the equine model, that is being not only an experimental animal but also a target animal with a clear and unmet clinical need.

### 2.9 Future research

At this time, our opinion is that there are two major avenues that could produce substantial progress in the attempts to restore function in a joint with acute or chronic osteochondral defects and in preventing development of osteoarthritis.

The first would be to create novel strategies based on the developmental biology of articular cartilage, mimicking the embryonic and fetal mechanisms that produce the tissue to begin with. Unfortunately, this ambitious goal remains elusive at the moment, mainly because currently there is poor understanding of the developmental biology of articular cartilage. It is still unclear how articular cartilage formation initiates in the embryo and how it is brought to completion and maturity in young adult life.^63^ Investing in more basic research would seem to be the most logic path to follow, although (at least in the short term) it is less economically appealing than seeking and finding a commercially usable product to fill osteochondral defects with.

**Figure 8:**
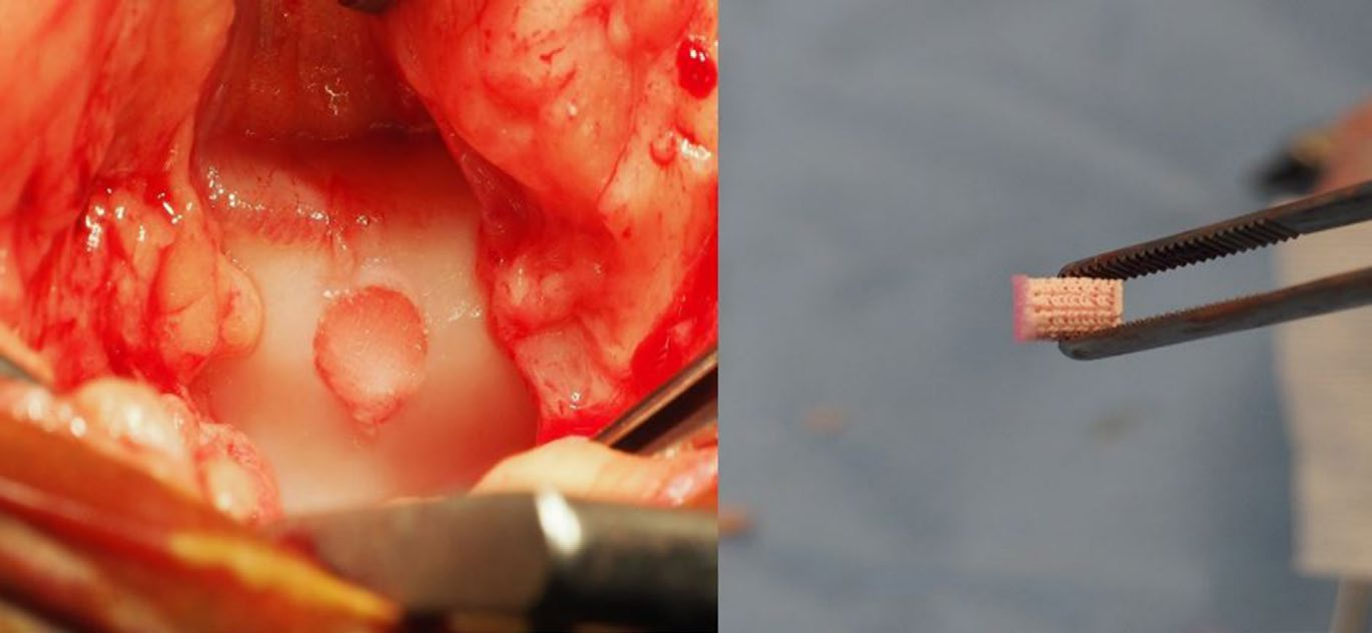
Examples of a biphasic scaffold. The bony portion of the osteochondral defect is filled with the ceramic scaffold, whereas the hydrogel which is previously cast and cross‐linked on top, fills the cartilage interface. These type of osteochondral plugs can be placed into created defects with a ‘press‐fit’ application. In the left image: osteochondral defect on the medial ridge of the femur of a horse, filled with a biphasic scaffold (right image)

The second way to improve therapeutic efficacy would be to focus attempts at cartilage repair more on functional results. Proteoglycan renewal can take up to 25 years in a joint whereas the half‐life of collagen is estimated to range from several decades to up to 400 years.^55^ The current paradigm of tissue engineering aims to create the right conditions for the production of new natural tissue.^64^ Perhaps, the focus should shift from the elusive goal of regeneration to achieving functional long‐term repair. In this light, the resorbable nature of the implant materials might be questioned and materials with half‐life that are similarly as long as those of the natural components of the extracellular matrix, for example polyurethane elastomers might be considered.^65^ The need for clinically and commercially viable methods promotes ‘simplification’ of the biological process of osteochondral repair. Strategies to achieve this include eliminating the need for two procedures, by utilising ‘press‐fit’ techniques with no additional need for fixation and by lowering regulatory hurdles by abstention of use of culture‐expanded cell populations (Figure 8).

In conclusion, we believe future research should focus on function of repair cartilage rather than on visual appearances or biochemical analyses. Mere production of collagen type II and glycosaminoglycans is not enough to create functional properties similar to those of native cartilage, as function is determined by the inseparable combination of constituting elements with a highly specific architectural arrangement.^66^ Future studies should always include assessment of repair cartilage structure and accurate biomechanical testing. In order to avoid disappointment when testing a successful short‐term result in long‐term studies,^31^, ^32^, ^37^, ^44^ short‐term studies should be regarded as pilot or proof‐of‐concept studies with a low number of animals and not pretend to provide clinically relevant outcomes. The duration of long‐term in vivo studies should be based on the reported time lapse after which clinical degeneration of repair cartilage begins. Continuous modification of repair tissue has been reported up to after 12 months in equine studies^14^ and 24 months in human studies with microfracture.^67^

Based on the current state of knowledge, treatment of experimentally created lesions on the lateral trochlea of the femur showed best results with the matrix‐assisted implantation of chondrocytes (MACI). Mosaicplasty technique showed best results on the medial condyle of the femur but not in the carpus. The justified concerns on the expense and high expertise level needed for these procedures make it clear how the optimal solution to repair of chondral and osteochondral defects in the horse is yet to be found. Nevertheless, our view is that the short‐term benefits (2‐3 years) of microfracture may still be of interest in the equine racing industry given the inexpensive and simple nature of the treatment.

## ETHICAL ANIMAL RESEARCH

No ethical approval needed.

## OWNER INFORMED CONSENT

Not applicable.

## AUTHOR CONTRIBUTIONS

M. C. Fugazzola designed and prepared the manuscript. P. R. van Weeren contributed with intellectual input and editing. M. C. Fugazzola had full access to all the data in the study and takes responsibility for the integrity of the data and the accuracy of the data analysis. Both authors have approved the final version of the manuscript.

## CONFLICT OF INTEREST

No competing interests have been declared.
