# Supplementary material for: Surgical osteochondral defect repair in the horse—a matter of form or function?
Source: Equine Vet J. 2020 Feb 19;52(4):489–99. doi: 10.1111/evj.13231 (PMC7317185; doi:10.1111/evj.13231)
Supplement: Supplementary file 1 [file EVJ-52-489-s001.pdf]

Equine Veterinary Journal BEVA

Supplementary Item 1: Overview of experimental osteochondral and chondral repair studies in the horse.

Experiment	No. of horses	Site of defect	Size of defect	Depth of defect	Study duration	Outcome parameters analysed	Type of tissue * formed/outcome	Reference
**Treatment:** Microfracture **Control:** empty defect	5	MFC and radial carpal bone	1 cm^2	Chondral full thickness	4 months	gross appearance, histology, histomorphometry, biochemistry	Fibrocartilage/ Increased volume of repair tissue in treated group	14
**Treatment:** Microfracture **Control:** empty defect	5	MFC and radial carpal bone	1 cm^2	Chondral full thickness	12 months	gross appearance, histology, histomorphometry, biochemistry	Fibrocartilage/ Increased volume of repair tissue in treated group	14
**Treatment:** Mosaicplasty **Control:** sound cartilage in same anatomical location	6	Radial facet of 3rd carpal bone	4.5 mm diameter	osteochondral	9 months	2nd look arthroscopy, gross appearance, histology, biochemistry	Significant decrease in GAG-content in all grafts, degeneration of cartilage in 6 out of 18 grafts	26
**Treatment:** Mosaicplasty **Control:** absent	6	MFC	6.0 mm diameter	osteochondral	12 months	2nd look arthroscopy, histology	5 out of 10 samples hyaline cartilage	27
**Treatment:** ACI **vs** CAIS **Control:** scaffold without cells	10	Lateral trochlear ridge of the femur	15 mm diameter	chondral full thickness	12 months	2nd look arthroscopy, gross appearance, histology, immunohistochemistry, biochemistry	Fibrocartilage	28
**Treatment:** ACI **Control:** periosteal flap alone	16	Lateral trochlear ridge of the femur	15 mm diameter	8 chondral full thickness 8 chondral partial thickness	2 months	gross appearance, histology, immunohistochemistry, biochemistry	Fibrocartilage/ Higher total histology scores for partial thickness lesions	30
**Treatment:** Fibrin/chondrocyte graft + IGF I **Control:** Fibrin/chondrocyte graft	8	Lateral trochlear ridge of the femur	15 mm diameter	chondral full thickness	8 months	gross appearance, histology, immunohistochemistry, biochemistry	Fibrocartilage	31
**Treatment:** Fibrin/chondrocyte graft **vs** Fibrin/IGF- 1 genetically transduced chondrocyte graft **vs** 0-	26	Lateral trochlear ridge of the femur	15 mm diameter	chondral full thickness	8 months	gross appearance, histology, immunohistochemistry, biochemistry, gene analysis	Not defined/ Higher total histology score of Fibrin/IGF- 1 genetically transduced chondrocyte graft **and** 0- gene-	32

gene-genetically transduced chondrocyte graft **Control:** fibrin alone							genetically transduced chondrocyte graft when **compared to** Fibrin/chondrocyte graft and fibrin alone	
**Treatment**: MACI **vs** collagen membrane alone **vs** empty defect **Control**: absent	9	Medial trochlear ridge of the femur	15 mm diameter	Chondral full thickness	12 months	gross appearance, histology, immunohistochemistry, biochemistry	Fibrocartilage/ Higher total histology score of MACI **compared to** empty defects and collagen membrane alone	33
**Treatment**: MACI **vs** collagen membrane alone **vs** empty defect **Control**: absent	6	Medial trochlear ridge of the femur	15 mm diameter	Chondral full thickness	18 months	gross appearance, histology, immunohistochemistry, biochemistry	Fibrocartilage/ Higher total histology score of MACI **compared to** empty defects and collagen membrane alone	33
**Treatment** MACI **Control**: empty defect	6	Lateral trochlear ridge of the femur	15 mm diameter	Chondral full thickness	6 months	2nd look arthroscopy, gross appearance, histology, immunohistochemistry, biochemistry, biomechanical testing	Fibrocartilage/ Higher total histology score of MACI **compared to** empty defects	34
**Treatment**: MACI **Control:** collagen membrane alone **and** empty defect	24	Lateral trochlear ridge of the femur	15 mm diameter	Chondral full thickness	13 months	2nd look arthroscopy, gross appearance, histology, immunohistochemistry, biochemistry, gene expression	Fibrocartilage Higher total histology score of MACI **compared to** empty defects **and** collagen membrane alone	35
**Treatment:** BMC + microfracture **Control:** microfracture	12	Lateral trochlear ridge of the femur	15 mm diameter	Chondral full thickness	8 months	2nd look arthroscopy, gross appearance, histology, immunohistochemistry, biochemistry	Not defined/ Higher mean histology score for BMC **compared to** microfracture alone	22
**Treatment:** BMC **Control:** microfracture	8	Lateral trochlear ridge of the femur	15 mm diameter	Chondral full thickness	12 months	2nd look arthroscopy, gross appearance, histology,	Fibrous tissue/ fibrocartilage	37

						immunohistochemistry, biochemistry		
**Treatment:** Fibrin containing MSC<br>**Control**: Fibrin only	6	Lateral trochlear ridge of the femur	15 mm diameter	Chondral full thickness	8 months	2nd look arthroscopy, gross appearance, histology, immunohistochemistry, biochemistry	Fibrocartilage	39
**Treatment:** APEF + MSC<br>**Control**: APEF alone	12	Lateral trochlear ridge of the femur	15 mm diameter	Chondral full thickness	12 months	2nd look arthroscopy, gross appearance, histology, immunohistochemistry, biochemistry, micro CT, biomechanical testing	Fibrocartilage with bone formation in 4 treated defects	40
**Treatment:** self-assembling peptide hydrogel + microfracture **vs** self-assembling peptide hydrogel alone<br>**Control:** empty defect **and** microfracture alone	32	Medial trochlear ridge of the femur	15 mm diameter	Chondral full thickness	12 months	2nd look arthroscopy, gross appearance, histology, immunohistochemistry, biochemistry, biomechanical testing	Fibrous tissue to fibrocartilage	41
**Treatment:** Scaffold + Microfracture + PRP<br>**Control:** Microfracture alone	5	Lateral trochlear ridge of the femur	10 mm diameter	Chondral full thickness	13 months	2nd look arthroscopy, gross appearance, histology, immunohistochemistry, biochemistry, Micro CT	Fibrocartilage/ higher total histology score with Biocartilage treatment **compared** to microfracture alone	42
**Treatment:** CRD<br>**Control:** Microfracture	12	Lateral trochlear ridge of the femur	10 mm diameter	Osteochondral and full thickness chondral	24 months	2nd look arthroscopy, gross appearance, histology, immunohistochemistry, biochemistry, biomechanical testing	Fibrocartilage	43
**Treatment:** CDM scaffold + CaP base for osteal part	8	Medial trochlear ridge	11 mm diameter	Osteochondral	6 months	2nd look arthroscopy, gross	Fibrocartilage	45

**Control:** CDM scaffold only		of the femur				appearance, histology, immunohisto chemistry, biochemistry, Micro CT, biomechanic al testing		

*Because various different histology scoring systems were used, the here presented type of tissue produced/outcome was based on the reported results of each study. The outcome column focusses only on histologic characterisation of repair tissue. For all other variables analysed, only significant differences between groups are mentioned.

Abbreviations: ACI = autologous chondrocyte implantation; CAIS = single step ACI: cartilage auto- graft implantation system; IGF-1 = Insulin- growth factor 1; BMC = bone marrow concentrate; MSC = mesenchymal stem cells; APEF = autologous platelet-enriched fibrin; CRD = biphasic cartilage repair device; CDM = cartilage-derived matrix scaffold; CaP = calcium phosphate; MFC = medial femur condyle
